# Spectrum of paediatric rheumatic diseases in Nigeria

**DOI:** 10.1186/s12969-017-0139-3

**Published:** 2017-01-31

**Authors:** Babatunde Hakeem Olaosebikan, Olufemi Oladipo Adelowo, Barakat Adeola Animashaun, Richard Oluyinka Akintayo

**Affiliations:** 1 Department of Medicine, Lagos State University Teaching Hospital, Ikeja, Lagos State Nigeria; 2 Department of Paediatrics, Lagos State University Teaching Hospital, Ikeja, Lagos State Nigeria; 3 Department of Medicine, University of Ilorin Teaching Hospital, Ilorin, Kwara State Nigeria

**Keywords:** Paediatric rheumatic disease, Nigeria, Juvenile idiopathic arthritis, Juvenile systemic lupus erythematosus

## Abstract

**Background:**

Paediatric rheumatology service in Sub-Sahara African is virtually not available as there is a shortage of paediatric rheumatologists and other rheumatology health professionals. We aim to describe the clinical spectrum and the frequencies of paediatric rheumatic diseases (PRDs) in Lagos State University Teaching Hospital (LASUTH), Lagos, Nigeria.

**Methods:**

This is a retrospective review of patients with PRDs seen over a five year period (March 2010 to February 2016) at the rheumatology clinic and children ward of LASUTH. We reviewed the folders of 57 patients from our records. The demographics, baseline laboratory features, clinical diagnosis, treatment patterns and patient outcomes were extracted and analyzed. Clinical and laboratory characteristics between patients with Juvenile idiopathic arthritis (JIA) and patients with juvenile connective tissue diseases (JCTD) were compared using Fisher’s exact test.

**Results:**

Fifty seven patients were studied with a female to male ratio of 3 to 1 (Female: 43; M: 14). The mean age at presentation in years was 14 ± 4.4 years (range: 1.5–22 years). The mean duration of symptoms before diagnosis was 18.4 ± .9 months (range: 2–60 months). The diagnostic types of PRDs included 28(49.1%) cases of JIA. These were made up of 14 cases of polyarticular JIA, nine cases of oligoarticular JIA and 5 cases of systemic onset JIA. Others were 18 (24.6%) cases of juvenile systemic lupus erythematosus (JSLE), 3 (5.3%) cases of joint hypermobility syndrome, 2 (3.5%) cases of juvenile systemic sclerosis, 2 (3.5%) cases of fibromyalgia, 2 (3.5%) cases of plantar fasciitis, 1 (1.6%) case of juvenile dermatomyositis (JDM), 1 (1.6%) case of juvenile polymyositis-systemic lupus erythematosus (PM-SLE) overlap, 1 (1.6%) case of secondary bilateral knee osteoarthritis from Blount disease, 1 (1.6%) case of secondary osteoporosis from childhood leukemia and 1 (1.6%) case of Osgood-Schlatter’s disease. Constitutional symptoms and extra-articular diseases were significantly more frequent among JCTD cases than among the JIA cases (Constitutional symptoms: 100% vs 83.3%, p = 0.003; extra-articular disease: 100% vs 10.7%, p = 0.001). The percentage mortality in this study was 10.5% while 20 (35.1%) of the patients were lost to clinic follow up.

**Conclusion:**

The pattern of PRDs observed in this study is similar to that described in South African and North American series but it differs from patterns reported in Asian series. Although hitherto largely unrecognized, PRDs may constitute a substantial cause of morbidity and mortality in black Africans.

## Background

The burdens and impacts of malnutrition, poverty-related and infectious diseases are well known in Sub-Sahara Africa. There is however, little or no attention to non-communicable diseases including paediatric rheumatic diseases (PRDs). The shortage of paediatric rheumatologists and other rheumatology health professionals as well as a general lack of awareness of this subject often contribute to missing these diagnoses and a consequent perception of rarity of PRDs in Africa [1]. In developing countries, prevalence estimates of PRDs are usually difficult to determine. This is especially so in Africa where access to pediatric rheumatology service is rare [2]. In Singapore, the prevalence of true PRDs among all cases referred to childhood rheumatology clinic is 57.8%, while in four Canada provinces, the prevalence estimates over 9 to 14 year period for systemic autoimmune rheumatic diseases (SARD) is 2 SARD cases per 10,000 residents aged 18 or less [3, 4]. However, this estimate did not include frequency of individual SARD. The frequencies and spectrums of PRDs vary in different studies from the western hemisphere and Asia. Whilst some studies from North America and Europe have reported JIA as the most frequent PRD, a study from Asia found systemic vasculitis as the most common [3, 5–8]. In Africa, there are few reports on PRD conditions such as JIA, juvenile systemic lupus erythematosus (JSLE), and juvenile dermatomyositis (JDM) [9–11]. However, there is a dearth of reports on the frequencies and spectrums of PRDs in various Sub-Saharan African settings [1]. A hospital-based report of the spectrum of PRDs in two tertiary institutions in South Africa has documented a relative frequency of 33.3% for JIA and 10.2% for juvenile connective tissue diseases (JCTD) [12]. Also, inflammatory arthritides were the most frequent musculoskeletal conditions among Kenyan children in outpatient clinics and wards [13, 14]. Patients with JCTD were, however, not included in the Kenyan studies. In a new rheumatology clinic in Zambia, Chipeta et al. registered 230 pediatric rheumatic cases over 2 years but the pattern of the PRDs was not stated in their report [15].

Epidemiological reports of PRDs in Africa are hampered by the lack of trained professionals in the field of rheumatology. Therefore, the burden of these diseases is unknown. Moreover, cases are often misdiagnosed or under-diagnosed and this is helped by a number of factors. One is the widespread belief that arthritis does not occur in children. Also, the much higher rate of infectious conditions often poses a lot of confusion and tends to lead to a delay in definitive diagnosis. Clinicians might believe they are dealing with an occult infection over a sustained period in a true case of rheumatic disease.

To the best of our knowledge, there have been no published reports of the spectrums of PRDs among West Africans. This is a retrospective study of the types and frequencies of PRDs in Nigerians attending a tertiary teaching hospital.

## Methods

This is a retrospective review of PRD cases managed between the first week of March 2010 and the last week of February 2016 at the adult rheumatology unit of LASUTH. LASUTH is a tertiary teaching hospital in the cosmopolitan city of Lagos. The hospital has one of the two adult rheumatology clinics in the city. There is no paediatric rheumatology clinic in Nigeria and as such pediatric rheumatic patients are managed by adult rheumatologists in conjunction with paediatricians. The paediatric patients with either musculoskeletal complaints or systemic symptoms suspicious of a rheumatic disease were referred for rheumatology review from the paediatric clinic, peripheral hospitals or paediatric wards. The diagnosis of PRDs was based on validated criteria such as the American College of Rheumatology (ACR) classification criteria, International League of Association of Rheumatology criteria, and other validated criteria for rheumatic diseases. The entry criterion for case selection was the diagnosis of a PRD in the patient’s record either as paediatric-onset disease in adult or a paediatric-age patient. Paediatric was defined as disease onset before the 16^th^ birthday in all cases except JSLE where disease onset before the 18^th^ birthday was adopted. The hospital records were retrieved for each patient and details of biodata, baseline clinical parameters, baseline laboratory indices, clinical diagnoses, drug treatments and patient outcomes were extracted. The duration of symptoms was taken as the onset from the first symptoms to clinical diagnosis at our facility. The full blood count, haematocrit, and erythrocyte sedimentation rate were documented in 50 cases (87.7%). Furthermore, C-reactive protein and serum ferritin were recorded for 37 cases (64.9%). Antinuclear antibody (ANA) was recorded in 48 cases(84.2%) while anti-extractable nuclear antigen (ENA) was documented in 16 patients(28.1%). Rheumatoid factor (RF) was recorded in seven patients (12.3%). ANA and ENA were analyzed by Enzyme-linked immunosorbent assay (ELISA) methods and RF was assayed by nephelometry.

### Statistical methods

Fisher’s exact test was applied to compare the clinical and laboratory characteristics between patients with JIA and patients with JCTD. All statistical analyses were done using the SPSS version 21(IBM USA, Armonk, NY 10504). A *p* value <0.05 was considered to be significant. Ethical approval was granted by the research and ethics committee of Lagos State University Teaching Hospital (LASUTH)

## Results

A total of 57 patients with PRDs, which included 11 cases diagnosed in pediatric ward, 30 referred cases from general paediatric clinic, and 16 patients referred from peripheral hospitals were managed at the adult rheumatology unit. These accounted for 2.4% of the 2330 rheumatic cases diagnosed and managed in our institution over the study period. As shown in Table 1, three quarters of our PRD cases were female and the mean age at presentation was 14 years. The mean duration of symptoms before the diagnosis was made was 18 months and musculoskeletal pain was documented in 52 (92%) PRD cases.

**Table 1:** Baseline demographic and clinical characteristics of 57 Nigerian children with PRDs

	n (%)	Range	Mean(±SD)
Female	43(75.4)		
Male	14(24.6)		
Age at presentation (years)		1.5–22	14(±4.4)
Duration of symptoms (months)		2–60	18.4(±1.9)
Musculoskeletal pain	52(91.2)		
Arthritis	40(70.2)		
Constitutional symptoms	33(57.9)		
Extra-articular disease	21(36.8)		

### PRDs in 57 Nigerian children

As shown in Table 2, JIA was the most frequent PRD seen in 28 (49%) of all cases. Polyarticular JIA(50%) was the most frequent of the three types of JIA observed. A total of 18 JCTD cases were seen. These accounted for 31.6% of all PRDs. They include 14 cases of JSLE, two cases of juvenile systemic sclerosis, and a case each of juvenile dermatomyositis and PM-SLE overlap.

**Table 2:** Spectrum of PRDs in 57 Nigerian children

Pediatric rheumatic diseases	Frequency (%)
Chronic inflammatory rheumatic diseases	
Juvenile idiopathic arthritis	28(49.1)
Systemic onset JIA	5(17.8)
Polyarticular JIA	14(50)
Oligoarticular JIA	9(31.2)
Juvenile connective tissue diseases (JCTD)	
JSLE	14(24.6)
Juvenile systemic sclerosis	2(3.5)
Juvenile dermatomyositis	1(1.6)
PM-SLE overlap	1(1.6)
Other rheumatic diseases	
Joint hypermobility syndrome	3(5.3)
Isolated plantar fasciitis	2(3.5)
Fibromyalgia	2(3.5)
Juvenile autoimmune uveitis	1(1.6)
Secondary osteoarthritis	1(1.6)
Secondary osteoporosis	1(1.6)
Osgood-Schlatter’s disease	1(1.6)

*JSLE* juvenile systemic lupus erythematosus, *PM-SLE overlap* polymyositis-systemic lupus erythematosus overlap

### Laboratory features

As shown in Table 3, 23 patients had positive ANA while 13 patients had positive ENA antibody. Anaemia was recorded in 37 PRD patients (74%) while rheumatoid factor was positive in two patients with polyarticular JIA. The mean haematocrit was 27.7% while the mean ESR was 72.9 mm/h.

**Table 3:** Laboratory characteristics of Nigerian children with PRDs

Laboratory abnormalities	Total number of PRD cases tested	n (%)
Anaemia(haematocrit < 33%)	50	37(74)
Elevated ESR(ESR > 20 mm/h)	50	42(84)
Elevated CRP(CRP > 8 mg/l)	37	32(86.4)
Elevatedferritin (Ferritin > 150 μg/l)	37	27(72.9)
Elevated RF(Rh-factor > 30iu/ml)	7	2(28.6)
Positive Anti-nuclear antibody	48	23(47.9)
Positive Anti-ENA antibody	16	13(81.2)
Laboratory parameters in SARDs	Mean(±SD)	Range
Haematocrit (%)	27.7(±7.5)	14–42
ESR (mm/h)	72.9(±37.4)	4–142
WBC (/μL)	11,081(±8,742)	1,350–45,600
Platelet count (/μL)	336,166(±191,938)	81,200–820,000
CRP (mg/L)	8.1(±4.9)	3–21
Ferritin(mg/dl)	548.5(±273.4)	62–2,140

*PRD* paediatric rheumatic disease, *ESR* erythrocyte sedimentation rate, *CRP* C - reactive protein, *ENA* Extractable nuclear antigen, *WBC* white blood cell. Normal reference range from our laboratory: Haematocrit-33-45%, ESR-0-20 mm/h, WBC-4500-13500/μL, Platelet count- 150,000-450,000/μL,CRP-0-8 mg/l, ferritin-10-150 μg/l

The JCTD cases seen include JSLE, juvenile systemic sclerosis, juvenile dermatomyositis, and PM-SLE overlap. In addition to JIA, these five diagnoses constituted the chronic inflammatory rheumatic disorders managed. Constitutional symptoms such as progressive weight loss, recurrent fever and fatigue were present in all patients with JCTD while constitutional symptoms were found in 15 (53.6%) patients with JIA (*p* < 0.001). Elevated ESR was found in 15 (83.3%) of JCTD patients and 25 (89.3%) of JIA patients. However, the difference in these proportions was not statistically significant (*p* = 0.666). Extra-articular manifestations included organ based features such as nephritis, pneumonitis and uveitis which excluded the musculoskeletal and constitutional symptoms; and they were found in all 18 (100%) patients with JCTD and 3 (10.7%) patients with JIA (*p* < 0.001).

### Treatment and outcome patterns in Nigerians with PRDs

Non-steroidal anti-inflammatory drugs (NSAIDs) were used for treatment in 40 (70.1%) cases while oral prednisolone was used in 32 (56.1%) cases. Twenty-five (43.8%) patients with JIA had methotrexate while all cases with JSLE had hydroxychloroquine. Five patients had biologic agents (rituximab in four and etanercept in one) out of 18 biologic-eligible patients. Rituximab was used in four JSLE patients with refractory lupus nephritis unresponsive to prednisolone, hydroxychloroquine, and azathioprine or mycophenolate mofetil while etanercept was administered in one refractory rheumatoid factor-positive polyarticular JIA patient. This latter patient happened to be the only survivor among all patients that had biologics. There were six mortalities accounting for 10.5% of the PRD cases while 20 (35.1%) patients were lost to follow up. Four mortalities were in patients with JSLE while two were in patients with systemic onset JIA. Progressive renal failure from lupus nephritis was the cause of mortality in the deceased JSLE patients. While one patient with systemic onset JIA died from overwhelming sepsis, the other died from suspected macrophage activating syndrome.

## Discussion

This is a report of patients with PRDs seen over a 5-year period by an adult rheumatology team. PRDs accounted for 2.8% of all inpatient and outpatient rheumatic cases managed over the period. This contrasts with the prevalence of 57.8% documented in childhood rheumatic clinic in Singapore [3]. This difference may be attributed to the primary adult focus of our unit. We noted that the mean age of presentation in our series is higher than the mean age reported among US patients with PRD (14 ± 4.4 years vs 7 ± 5.76 years) [16]. Besides, Nigerian patients with PRDs have high mean duration of symptoms before diagnosis (18.2 months). Late presentation and delayed diagnosis may be due to poverty, ignorance, and inadequate healthcare infrastructures and personnel. The inclusion of patients older than 18 years with retrospective diagnoses of childhood-onset diseases may also explain the high mean age of presentation observed in this series. There are no paediatric rheumatology clinics or paediatric rheumatologists in Nigeria. However, delay in diagnosis was also documented in the USA, a country with about 300 certified pediatric rheumatologists [17]. The report stated that 60% of children referred for suspected rheumatic conditions did not have confirmed rheumatic diagnosis and further rheumatologic follow up was requested in 40% of cases. The complex tendencies of many JCTD may inherently present diagnostic difficulties and sometimes prolong the period of evaluation before a diagnosis is made. Moreover, the near similar manifestations of JCTD with common childhood infections and malignancies may lead to initial diagnostic confusions in lower hospitals contributing to the delay in presenting for rheumatologist care.

We observed that PRDs are more frequent among female patients than in the male patients. This observation may be due to the substantial proportion of JCTD and JIA cases in our study as these conditions are generally more common in the female than in the male [4]. The most frequent clinical feature among our patients was musculoskeletal pain occurring in 91.2% while constitutional symptoms were recorded in 57.9% of PRD cases. Similarly, it was observed in Canada that musculoskeletal pain was the most common presenting complaint in a children rheumatology clinic [17]. In addition, they found that joint swelling and fever were most predictive of the need for further rheumatology follow up in their clinic.

JIA is the most frequent PRD condition identified in this study accounting for 49.1% of PRD cases. This is followed by JSLE in 24.6% of the study subjects and JCTD, on the whole, constituted 36.1% of PRD subjects. This result is in consonance with various studies from Europe, South Africa and USA that documented JIA as the most frequent PRD while either juvenile lupus or all CTDs was the next in their reports [12, 16, 18]. Previous reports from Africa suggest that JIA, JSLE, and JDM may not be uncommon in Sub-Saharan Africa [9, 10, 19]. Systemic vasculitis was the commonest PRD observed in Singapore by Tan et al. and it was found in a low proportion (4.3%) in a South African series but we did not document any systemic vasculitis in our series [3, 12]. Geographical variations in the frequencies of vasculitides are likely to be the reason for this finding. We found a low proportion of non-inflammatory rheumatic conditions such as joint hypermobility syndrome, isolated plantar fasciitis, fibromyalgia, and secondary osteoarthritis in our series. Orthopedic surgeons in our hospital often manage children with mechanical and non-inflammatory PRDs. This may explain the low frequency of this category of cases in this study.

Patients with a high clinical likelihood of a particular PRD had laboratory investigations tailored to that condition. This policy was adopted in our center to reduce the cost and burden of investigations on our patients as most of these patients pay directly for their laboratory investigations. The proportion of PRD cases with anaemia and elevated ESR in this study is high. This is due to a high proportion of subjects with SARDs in this study. Although, the proportions of nutritional anaemia and occult parasitaemia were not estimated in our patients, they may contribute to the high percentage of subjects with anaemia in our study.

We face enormous challenges in Africa with regards to anti-rheumatic medications. In Nigeria, numerous NSAIDs and oral prednisolone are readily available over the counter and patients often self-medicate with them before seeking a physician’s review. Moreover, some synthetic disease-modifying anti-rheumatic drugs (DMARDs) are not readily available and when they are available, they may be unaffordable for many patients. Doctors and patients sometimes source for DMARDs from other countries often at prohibitive costs. Standard therapy with NSAIDs, DMARDs and glucocorticoids were administered to the majority of our patients with chronic inflammatory rheumatic diseases. Access to biologic DMARDs is limited in most African countries and this often limits the quality of specialist care a patient could get [20]. The highly limited access to biologics is a problem in the developing world where patients often fail to get access to specialist care until late in their disease. Rituximab and Etanercept, the only biologic DMARDs available in Nigeria, are largely unaffordable for our patients who have to pay for their treatment.

JSLE contributed four out of the six deaths and all these four patients had lupus nephritis unresponsive to standard therapy. Also, they had rituximab infusions in addition to other supportive care. However; they died from progressive renal failure after haemodialysis was instituted. The other two patients had systemic JIA and the causes of death in them were overwhelming sepsis and suspected macrophage activating syndrome. Systemic lupus erythematosus has been shown to run a more severe and more aggressive course in blacks and, even worse, in children [21]. Long delays before the diagnoses were made and inadequate treatments due to financial difficulties may have contributed to the high short-term mortality. In addition, some studies have suggested that socio-demographic disparities may influence the outcomes of these younger subjects [21, 22]. The proportion of PRD cases lost to follow up in this study was 35.1%. This high rate of discontinuation of care plan reflects the common pattern of unsustained treatment adherence among our patients. Poverty, illiteracy and various health related cultural beliefs may contribute to this phenomenon. Our study is also limited by a small sample size and the retrospective nature of the design. The burden of PRDs may have been under-appreciated due to the adult focus of our practice as there is no paediatric rheumatologist in our country. Furthermore, high default rate among our patients makes the actual treatment outcome difficult to determine. Prospective cohort studies on this subject will help to determine the course of PRDs and their outcomes among Nigerians.

## Conclusions

The spectrum of PRDs observed in this Nigerian series shares some similarities with patterns described in earlier studies from South Africa, North America and Europe. In all of these studies JIA accounts for the largest number of PRDs. Our patients with JIA tend to be younger than the patients presenting with JCTDs. However, systemic vasculitides, the most frequent PRDs in Singapore, were not observed in our series. The Nigerian patients with PRDs are more likely to be female, are more likely to present to the hospital later than children from the West and are more likely to present with musculoskeletal complaints than other symptoms.

## Abbreviations

ACR: American College of Rheumatology
ANA: Antinuclear antibody
DMARD: Disease-modifying anti-rheumatic drug
ELISA: Enzyme-linked immunosorbent assay
ENA: Extractable nuclear antigen
JCTD: Juvenile connective tissue diseases
JDM: Juvenile dermatomyositis
JIA: Juvenile idiopathic arthritis
JSLE: Juvenile systemic lupus erythematosus
JSLE: Juvenile systemic lupus erythematosus
LASUTH: Lagos State University Teaching Hospital
NSAID: Non-steroidal anti-inflammatory drug
PM-SLE overlap: Polymyositis-systemic lupus erythematosus overlap
PRDs: Pediatric rheumatic diseases
RF: Rheumatoid factor
SARD: Systemic autoimmune rheumatic diseases

## References

[1] Scott C, Webb K (2014). Paediatric rheumatology in sub-saharan africa. Rheumatology (Oxford).

[2] Lawrence RC, Helmick CG, Arnett FC, Deyo RA, Felson DT, Giannini EH (1998). Estimates of the prevalence of arthritis and selected musculoskeletal disorders in the united states. Arthritis Rheum.

[3] Tan JHT, Fun HS, Arkachaisri T (2012). Paediatrics rheumatology clinic population in singapore: The kkh experience. Proc Singap Healthc.

[4] Shiff NJ, Lix LM, Joseph L, Duffy C, Tucker LB, Svenson LW (2015). The prevalence of systemic autoimmune rheumatic diseases in canadian pediatric populations: Administrative database estimates. Rheumatol Int.

[5] Rosenberg AM (2005). Longitudinal analysis of a pediatric rheumatology clinic population. J Rheumatol.

[6] Sawhney S, Magalhães CS (2006). Paediatric rheumatology—a global perspective. Best Pract Res Clin Rheumatol.

[7] Malleson P, Fung M, Rosenberg A (1996). The incidence of pediatric rheumatic diseases: Results from the canadian pediatric rheumatology association disease registry. J Rheumatol.

[8] Bowyer S, Roettcher P (1996). Pediatric rheumatology clinic populations in the united states: Results of a 3 year survey. Pediatric rheumatology database research group. J Rheumatol.

[9] Adelowo OO, Umar A (2010). Juvenile idiopathic arthritis among nigerians: A case study. Clin Rheumatol.

[10] el-Garf A, Salah S (1990). Juvenile systemic lupus erythematosus among egyptian children. J Rheumatol.

[11] Adelowo O, Nwankwo M, Olaosebikan H (2014). Juvenile dermatomyositis in a nigerian girl. BMJ Case Rep.

[12] Okong’o LO, Scott C (2014). The spectrum of paediatric rheumatic diseases in two tertiary centres in cape town, south africa. Pediatr Rheumatol.

[13] Broten L, Migowa A, Scuccimarri R, Ng'ang'a E, Wachira J, Ngwiri T, et al. Spectrum of outpatient musculoskeletal visits at the largest pediatric center in east africa in 2011. Arthritis Rheum. 2013;65:S930–S1.

[14] Migowa A, Colmegna I, Wachira J, Ngwiri T, Hitchon C, Bernatsky S, et al. Spectrum of musculoskeletal inpatient diagnoses at the largest pediatric center in east africa in 2011. J Rheumatol. 2015;42:1271.

[15] Chipeta J, Njobvu P, McGill PE, Bucala R (2014). Progress made towards enhancement of rheumatology education and practice in zambia: Review of an ilar-supported project. Clin Rheumatol.

[16] Mahajan M, Shah M, Toth M, Mcninch N, El-Hallak M. Inpatient pediatric rheumatic diseases: Characteristics, cost and trends. Arthritis Rheum. 2014;66:S992.

[17] Rodriguez M, Ranieri D, Onel K (2016). Paediatric rheumatology referral patterns: presenting complaints of new patients at large, urban academic centre. Paediatr Rheumatol.

[18] Huemer C, Huemer M, Dorner T, Falger J, Schacherl H, Bernecker M (2001). Incidence of pediatric rheumatic diseases in a regional population in austria. J Rheumatol.

[19] Faller G, Mistry BJ, Tikly M (2014). Juvenile dermatomyositis in south african children is characterised by frequent dystropic calcification: A cross sectional study. Pediatr Rheumatol.

[20] Maini M, Adelowo F, Saleh J, Weshahi Y, Burmester G-R, Cutolo M (2015). The global challenges and opportunities in the practice of rheumatology: White paper by the world forum on rheumatic and musculoskeletal diseases. Clin Rheumatol.

[21] Son MBF, Johnson VM, Hersh AO, Lo MS, Costenbader KH (2014). Outcomes in hospitalized pediatric patients with systemic lupus erythematosus. Pediatrics.

[22] Levy DM, Peschken CA, Tucker LB, Chédeville G, Huber AM, Pope JE (2013). Influence of ethnicity on childhood‐onset systemic lupus erythematosus: Results from a multiethnic multicenter canadian cohort. Arthritis Care Res.

